# A Call for Transformation: Moving Away from Coercive Measures in Mental Health Care

**DOI:** 10.3390/healthcare11162315

**Published:** 2023-08-17

**Authors:** Lene Lauge Berring, Eugenie Georgaca

**Affiliations:** 1 Psychiatric Research Unit, Psychiatry Region Zealand, 4200 Slagelse, Denmark; 2 Department of Regional Health Research, University of Southern Denmark, 5230 Odense, Denmark; 3 School of Psychology, Aristotle University of Thessaloniki, 54124 Thessaloniki, Greece; georgaca@psy.auth.gr

Coercion is common practice in mental health care. Although the use of coercive measures is strictly regulated by legislation [1], coercive practices continue to be applied frequently in mental health services [2,3]. These practices vary significantly among European countries [4], indicating the role of local and national variations in mental health policies and practices.

The most commonly implemented formal coercive measures include involuntary hospitalization; observation; seclusion; forced medication; mechanical, chemical, and physical restraint; as well as compulsory treatment in the community [1]. Moreover, many informal coercive measures are part of everyday mental health practice that are not usually recognised as coercive and, therefore, are not researched and regulated. Such methods include locked doors, restrictions on using one’s phone, contacting others, receiving visitors [5,6], as well as using persuasion, leverage, and threats to regulate service users’ behaviours [7,8].

Coercive practices raise human rights issues, as they restrict the human rights of those subjected or exposed to them, and they violate ethical principles of professional practice, such as respect for a patient’s autonomy, beneficence, nonmaleficence, and justice [9]. The restriction, and often violation, of human rights during coercive practices is a major concern. The use of coercive measures compels behaviour against patients’ wishes, cancelling their autonomy, agency, and self-determination. This is incompatible with the United Nations Convention on the Rights of Persons with Disabilities or the values of any democratic society [10]. Given the ethical duties of mental health professionals to safeguard patients’ human rights, actively encouraging and supporting policies and practices that would reduce and end coercion in mental health care should be a primary concern of mental health professionals and policy makers, as affirmed by the Human Rights Commissioner Dunja Mijatović in 2019 [11]. This commitment obligates us to re-think how mental health services treat individuals with mental health conditions.

There is increasing evidence that coercive practices are experienced as dehumanizing and traumatic by service users, caregivers, and professionals [9,12]. Many research studies document the negative, often long-lasting, impact of coercion on those subjected or exposed to it [13,14] that has adverse consequences for service users’ engagement with mental health services and adherence to treatment [1], ultimately affecting their mental health in a detrimental way and hindering their recovery [15].

There is an increasing global impetus to replace these coercive measures with voluntary, consensual, and collaborative professional practices, thereby pre-empting, preventing, and, finally, ending the use of coercion in mental health systems [16]. This Special Issue of Healthcare strives to showcase research studies that contribute to our understanding of this crucial matter and signal the necessity for change at multiple levels.

Research-based evidence underscores that no single intervention can avoid the continued use of coercive measures [12]. This calls for a comprehensive strategy that addresses organizational factors, staff training, risk assessment, and environment and includes interventions like psychotherapy, debriefing, and advance directives. A focus on Trauma Informed Care [17], where the emphasis is on patients’ needs rather than their behaviours, could be a step towards preventing coercion. Assertive, community-based initiatives [18] can also reduce forced admissions, fostering a more supportive environment for people with severe disorders. Interventions such as the Six Core Strategies [19], which have been successfully implemented in the U.S., Finland, and the U.K. [20], and models like Safewards [21,22] have shown promising results in reducing conflict and the use of restrictive practices in psychiatric settings.

Dealing with the aftermath of being subjected to coercion requires a comprehensive response, involving person-centred therapeutic strategies, like cognitive-behavioural therapy and trauma-informed therapy, to help persons subjected to coercion process their experiences and rebuild resilience. Implementing post-incident review processes can generate valuable insights for understanding a coercive event and preventing future ones, whereas the use of advance directives can restore patient autonomy and facilitate healing [23].

In order to achieve a human rights-based approach in mental health care, it is critical to understand the link between the initiatives taken by the government and mental health authorities and their practical applications [24]. It is only through consultation with stakeholders, especially those with lived experiences, that we can generate informed recommendations for future prevention initiatives [2]. This transformation towards a recovery-oriented practice requires changes at multiple levels, including knowledge, attitudes, and practices of professionals, families, and users of the mental health care system [25]. This engagement can disrupt traditional approaches and perspectives, opening the way for new, more effective strategies. Frontline workers’ concerns about their safety must be considered alongside patients’ needs for dignity and autonomy.

Moving away from coercion in mental health services requires understanding the risk factors for coercion at individual, systemic and societal levels, especially through examining clinical practices and legal frameworks across countries. It also necessitates highlighting the impact of coercive practices for all involved as well as the best practices for minimizing that impact and supporting post-incident recovery. We also need to increase knowledge of alternative interventions and to demonstrate their effectiveness in preventing use of coercion and promoting recovery [26]. By including papers on all the topics above, this Special Issue aims to help form a more comprehensive understandings of the factors and processes that sustain the continued use of coercion in mental health care, in order to inform strategies for its prevention. It also aims to showcase innovative interventions implemented to date that may prevent or reduce the use of coercion or mitigate its impact on those implicated in it. The Special Issue is deliberately inclusive and comprehensive, consistent with a psychosocial, recovery-oriented approach to mental health; we aim to present empirical studies and systematic reviews from researchers and practitioners in all mental health related disciplines that reflect the perspectives of service users, practitioners, careers, and health service managers.

This Special Issue is related to and intends to disseminate the work of the Fostering and Strengthening Approaches to Reducing Coercion in European Mental Health Services (FOSTREN) Network. FOSTREN is a COST-funded action set up to establish a sustainable, multidisciplinary network of researchers and innovators who are committed to improving understanding on how to reduce coercion in mental health services. FOSTREN aims to summarise current knowledge on the most effective methods for the implementation and transformation of health services, as it relates to reducing coercion in mental health services. Specifically, it aims to advance understanding of the processes underlying the use of coercion and to examine successful interventions for reducing coercion in European mental health services. More importantly, FOSTREN’s main goal is to use this new understanding as a basis for setting best practice standards and recommendations and to disseminate them to policy makers and practitioners throughout Europe, aiming to develop mental health policies and practices that would reduce coercion, improve mental health care, and promote recovery.

Finally, this Special Issue of Healthcare is a call to action, an urging for a paradigm shift in mental health care, towards prioritizing patients’ rights, dignity, and well-being. Let us take the lessons from the research presented in this issue and the experiences of those on the front lines of mental health care to heart and strive to reduce and ultimately eliminate the use of coercive measures in our practices. Through collective action, we can bring about a future in which mental health care is more humane, ethical, and supportive of recovery.
